# Primary thyroid squamous cell carcinoma diagnosed with ^18^F-FDG PET/CT: a case report

**DOI:** 10.3389/fonc.2024.1434811

**Published:** 2024-07-08

**Authors:** Taiping Liao, Yongjun Long, Lingxiao Li, Qinlin Qi, Li Li, Guoxu Fu

**Affiliations:** Department of Nuclear Medicine, The Third Hospital of Mianyang (Sichuan Mental Health Center), Mianyang, China

**Keywords:** 18F, FDG, PET/CT, thyroid, squamous cell carcinoma

## Abstract

Primary thyroid squamous cell carcinoma is extremely rare. We report a case of primary thyroid squamous cell carcinoma diagnosed using ^18^F-FDG PET/CT. The patient presented with left axillary lymphadenopathy as the initial symptom. Fine-needle aspiration of the axillary lymph nodes indicated metastatic squamous cell carcinoma. To identify the primary tumor, the patient underwent an ^18^F-FDG PET/CT scan, which revealed a mass in the thyroid and multiple enlarged lymph nodes with abnormal FDG uptake. Pathological examination of the axillary lymph nodes and thyroid biopsy confirmed the diagnosis of primary thyroid squamous cell carcinoma with lymph node metastasis.

## Introduction

Primary thyroid squamous cell carcinoma is extremely rare, with an incidence rate of less than 1% (1), and it is a subtype of anaplastic thyroid carcinoma (2). It mostly occurs in elderly males (3), progresses rapidly, and has a very poor prognosis, with a survival period mostly shorter than one year (4, 5). In this case, we describe a rare instance of primary thyroid squamous cell carcinoma with multiple systemic lymph node metastases.

## Case presentation

**Figure 1:**
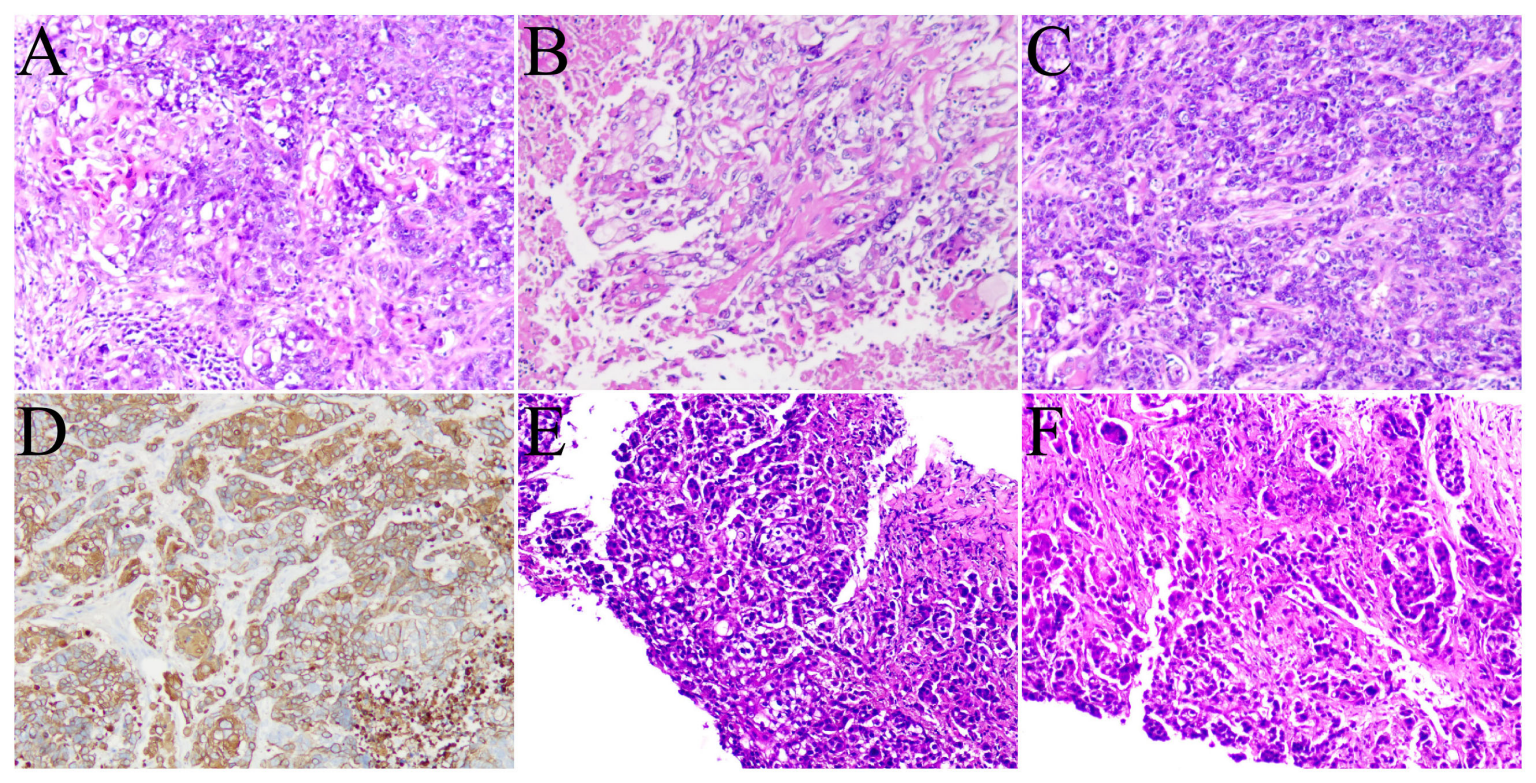
Images **(A–D)** show the pathological pictures of the left axillary lymph node biopsy. Images **(E, F)** show the pathological pictures of the thyroid biopsy.

Nine months ago, the 74-year-old male patient discovered a lump in the left axilla and did not pay much attention to it. Recently, the lump has gradually enlarged and become painful.One week ago, the patient visited our hospital. Ultrasound and CT scans revealed multiple enlarged lymph nodes in the neck and left axilla. Physical examination confirmed multiple enlarged lymph nodes in the neck, which were hard, poorly mobile, and mildly tender (**Figures 1A–D**). Fine-needle aspiration of the left axillary lymph nodes indicated metastatic squamous cell carcinoma, Immunohistochemistry results indicate: PCK (+), P40 (+), P63 (+), CD56 (+), PAX-8 (-), TTF-1 (-), CgA (-), S100 (-), NUT (-), P53 (+), Ki67 (+, 60%), EBER1/2-ISH (-). The tumor marker test results for this patient are as follows: SCC 5.13 ng/mL (reference range: 0-1.8 ng/mL), CYFRA 21-1 8.42 ng/mL (reference range: 0-3.30 ng/mL), NSE 28.70 ng/mL (reference range: 0-16.5 ng/mL), CA125 82.10 U/mL (reference range: 0-30.2 U/mL). The levels of CA199, CA724, AFP, and CEA are within normal limits. The patient has a history of appendectomy and a 10-year history of coronary artery disease.

**Figure 2:**
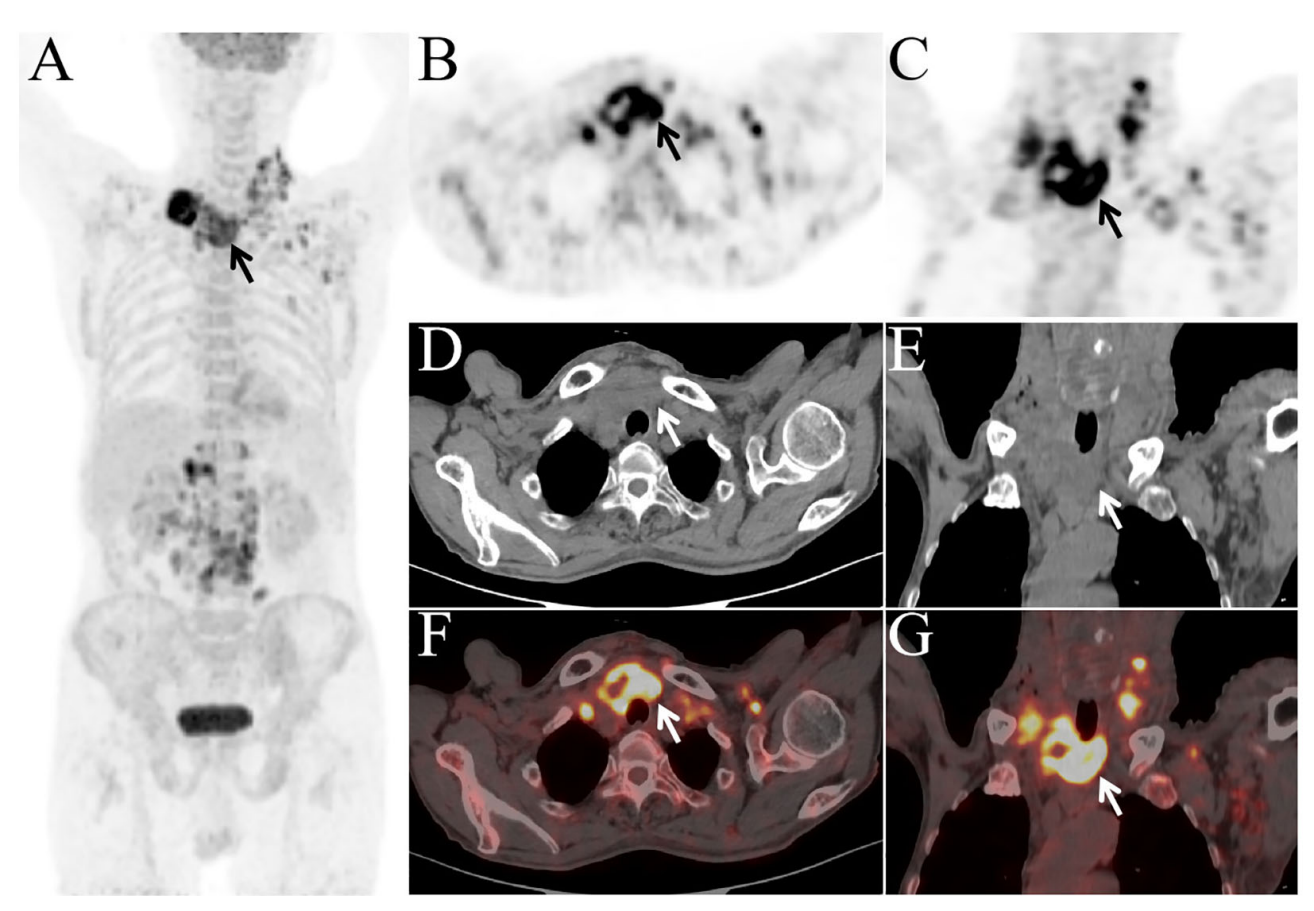
Whole-body imaging **(A)** revealed multiple areas of increased FDG metabolism in the thyroid region (arrow), neck, and abdomen. The axial and coronal images **(B, C)**, axial and coronal view of PET; **(D, E)**, axial and coronal view of CT; **(F, G)**, axial and coronal view of fusion images) showed a mass in the thyroid with increased FDG metabolism [**(B–G)**, arrow].

To identify the primary tumor, the patient underwent ^18^F-FDG PET/CT at our hospital. The PET/CT images showed a heterogeneous mass in the thyroid with significantly increased FDG uptake (**Figures 2A–G**, arrow), with an SUVmax of 10.0. Multiple enlarged lymph nodes in the neck, right supraclavicular fossa, left axilla, and abdomen,with an abnormally high FDG uptake, with an SUVmax of 15.3. No abnormal morphology or FDG metabolism was observed in other organs. Subsequently, the patient underwent fine-needle aspiration biopsy of the thyroid (**Figures 1E, F**), which revealed cancer cells with immunohistochemistry positive for P40. Based on the ^18^F-FDG PET/CT and pathological findings, the patient was diagnosed with primary thyroid squamous cell carcinoma with multiple systemic lymph node metastases. The patient is currently undergoing the first cycle of chemotherapy with paclitaxel and carboplatin.

## Discussion

Primary thyroid squamous cell carcinoma is a malignant tumor originating from thyroid tissue. In the latest World Health Organization classification of endocrine and neuroendocrine tumors, primary thyroid squamous cell carcinoma is included in anaplastic thyroid carcinoma,because squamous cell carcinoma of the thyroid often displays BRAF p.V600E mutations (87%) and exhibits positive immunohistochemical staining for the follicular cell markers PAX8 (91%) and TTF1 (38%) (6). Its incidence is extremely low, accounting for less than 1% of thyroid malignancies. Patients are mostly elderly males. However, it exhibits a high degree of malignancy, progresses rapidly, and carries a poor prognosis, with most patients surviving less than one year. ^18^F-FDG PET/CT can simultaneously display the glucose metabolism level and morphological information of lesions (7), which is valuable in the diagnosis and staging of thyroid cancer (8). There are few reports on ^18^F-FDG PET/CT imaging of primary thyroid squamous cell carcinoma (9, 10).

In this case, ^18^F-FDG PET/CT was crucial in distinguishing primary thyroid squamous cell carcinoma from secondary squamous cell carcinoma. Secondary squamous cell carcinoma could originate from other squamous cell carcinomas outside the thyroid, such as those in the lungs, esophagus, or head and neck region. By showing increased FDG uptake in the thyroid gland and the absence of abnormal FDG uptake in other common sites of squamous cell carcinoma, ^18^F-FDG PET/CT helped exclude the possibility of a metastatic origin. Additionally, the morphological information provided by PET/CT supported the localization of the tumor to the thyroid. Combined with pathological results showing squamous differentiation in the thyroid tissue, the diagnosis of primary thyroid squamous cell carcinoma was confirmed.

## Conclusion

Primary thyroid squamous cell carcinoma is extremely rare,this case emphasizes the value of ^18^F-FDG PET/CT in identifying primary tumor lesions and is also of great value in distinguishing between primary and secondary thyroid squamous cell carcinoma,which is of great significance for the diagnosis and staging of primary thyroid squamous cell carcinoma.

## Data availability statement

The original contributions presented in the study are included in the article/supplementary material. Further inquiries can be directed to the corresponding author.

## Ethics statement

The studies involving humans were approved by Ethics Committee of The Third Hospital of Mianyang. The studies were conducted in accordance with the local legislation and institutional requirements. The participants provided their written informed consent to participate in this study. Written informed consent was obtained from the individual(s) for the publication of any potentially identifiable images or data included in this article.

## Author contributions

LT: Writing – original draft, Writing – review & editing. LY: Writing – review & editing. LXL: Writing – review & editing. QQ: Writing – review & editing. LL: Writing – review & editing. FG: Writing – review & editing.
